# Exploring shared and unique benefits of passive and active prenatal intervention protocols on maternal wellbeing and neonatal outcomes: a combined quali-quantitative approach

**DOI:** 10.3389/fpsyg.2025.1553946

**Published:** 2025-04-29

**Authors:** Martina Arioli, Alessandra Consales, Melissa Savoldi, Ilenia Mastroianni, Maria Lorella Gianni, Lorenzo Colombo, Niccolò Giovannini, Chiara Sacchi, Viola Macchi Cassia

**Affiliations:** ^1^Department of Psychology, University of Milano-Bicocca, Milan, Italy; ^2^Department of Clinical Sciences and Community Health, Dipartimento di Eccellenza 2023-2027, University of Milan, Milan, Italy; ^3^NICU, Fondazione IRCCS Ca' Granda Ospedale Maggiore Policlinico, Milan, Italy; ^4^Department of Obstetrics and Gynecology, Fondazione IRCCS Ca’ Granda Ospedale Maggiore Policlinico, Milan, Italy; ^5^Department of Developmental Psychology and Socialization, University of Padova, Padua, Italy

**Keywords:** prenatal interventions, maternal mental health, perinatal outcomes, active prenatal protocol, passive prenatal protocol, thematic analysis, attachment, anxiety and depression

## Abstract

**Introduction:**

In the present study, the shared and distinct effects of two novel prenatal intervention protocols implemented during the last trimester of gestation on perinatal maternal wellbeing and perinatal outcomes were explored.

**Methods:**

A total of 250 pregnant women at 28 weeks gestation were randomly assigned to either a Passive relaxation-based Protocol or an Active Protocol involving active maternal engagement in various motor activities during music listening. Anxiety and depression symptoms and attachment to the foetus/infant were assessed through self-report questionnaires before (t0) and after (t1) the protocol, as well 1.5 months postpartum (t2). Qualitative data on participants’ emotional experiences while performing the activities were collected through weekly diaries, together with adherence to the intervention protocol and the amount of engagement with the prescribed activities.

**Results:**

Group-level analyses showed that both protocols were associated with improvements in anxiety symptoms and attachment to the foetus/infant that extended to the postpartum period, while a different picture emerged for depression symptoms. Analyses of the data collected through the diaries revealed a more nuanced picture, showing that, within each group, maternal wellbeing outcomes and/or emotional experiences during the protocol were modulated by participants’ adherence and engagement with the prescribed activities. No differences between the two intervention groups were found in terms of the perinatal outcomes considered, but the number of relaxation sessions positively predicted newborns’ gestational age.

**Discussion:**

These findings offer valuable insights into the mechanisms underlying the effects of passive versus active prenatal interventions, and point to the importance of tailoring intervention strategies based on individual preferences and perinatal stage.

## Introduction

Pregnancy is a transformative period marked by significant physical, psychological, and social changes that make expectant parents particularly susceptible to the influence of favourable or unfavourable conditions that accompany the critical transition to parenthood. During pregnancy, labour, and the postpartum period prospective parents, particularly women, experience a range of physiological and psychological shifts (Hart and McMahon, 2006), that impact their sense of self, purpose, and overall functioning. Physically, the body undergoes pregnancy-related changes to accommodate the growing foetus and prepare for childbirth. Emotionally and socially, individuals may experience shifts in their roles and relationships as they transition into the parental role. These changes, while challenging, also offer opportunities for personal growth as individuals navigate new roles and responsibilities while facing new potential stressors. Ultimately, it is the combination of experiences across the physical, physiological, psychological, and social spheres that shapes prospective parents’ wellbeing during the perinatal period, their developmental transition to parenthood and their readiness to engage in caring behaviours to ensure the survival of the infant (Feldman, 2015; Sacchi et al., 2021).

As such, research documented a strong and reciprocal connection between women’s wellbeing during pregnancy and pregnancy and postpartum outcomes, including the quality of bonding and emerging caregiving function. Globally, 15–25% of pregnant women experience anxiety or depressive symptoms (Jha et al., 2018; Fawcett et al., 2019), with these percentages being higher in lower-and middle-income countries (Dadi et al., 2020). Pregnant women typically report to experience twice the rate of depression symptoms compared to non-pregnant women (Ko et al., 2012), and these symptoms can negatively impact perinatal health in terms of greater obstetric complications (Huang et al., 2017) and elective caesarean section delivery (Sun et al., 2019). Similarly, the emotional bond the women form with her unborn baby during pregnancy (i.e., prenatal attachment) through engagement in thoughts, feelings and behaviours conveying caring, commitment, and interaction with the child (Salisbury et al., 2003) is negatively associated with perinatal anxiety and depression symptoms (e.g., McFarland et al., 2011; Özdemir et al., 2020) and poor pregnancy-related health practices (Lindgren, 2001). Recent research has also documented that maternal health and wellbeing during pregnancy can significantly impact the long-term health of the offspring through mechanisms of ‘foetal programming’. Foetal programming describes the process by which the foetus’ response to the intrauterine environment produces structural and functional changes in cells, tissues, and organ systems, which have long-term consequences for future health and disease susceptibility (Langley-Evans, 2006; Langley-Evans, 2015). Research conducted within the Developmental Origins of Health and Disease (DOHaD) research domain (Gluckman et al., 2016) has shown that environmental stressors linked not only to maternal malnutrition and overnutrition, infectious agents or substance abuse, but also to mental health issues and psychosocial stress that can lead to altered physiological and metabolic processes in the developing foetus that increase postnatal vulnerability to various physical and mental health problems. Accordingly, besides affecting physical health of pregnant women and postnatal maternal wellbeing, prenatal anxiety and psychological distress have been found as predictive factors for delayed motor and mental development in infancy (e.g., Huizink et al., 2003). Furthermore, newborns of mothers with prenatal depression or anxiety have a higher risk of being born prematurely or having a low birth weight (Accortt et al., 2015), which in turn are important risk factors for later infant health risks (e.g., Pusdekar et al., 2020; Diabelková et al., 2022).

In light of this evidence, boosting maternal psychological wellbeing in pregnancy appears as a promising strategy for mother-infant dyadic health prevention. Indeed, it might reduce mental health risks and support the transition to parenthood over pregnancy and postpartum, while simultaneously enhancing the environment for foetal development. For instance, mindfulness interventions have shown promise in improving depressive symptoms in low-risk pregnant women (Min et al., 2023), and physical activity during pregnancy has been shown to reduce the risk and severity of prenatal depression and anxiety, while also reducing stress levels and improving overall quality of life (Cai et al., 2022). Recent research is providing mounting evidence on the potential benefit of prenatal intervention programmes, defined as sets of specific activities engaged in by pregnant women aimed at promoting foetal wellbeing, for promoting maternal mental health and infant development. These programmes vary widely in structure and format, encompassing a diverse range of activities, spanning from relaxation techniques (e.g., Urech et al., 2010) and passive music listening (e.g., Çatalgöl and Ceber Turfan, 2022), to singing lullabies (e.g., Carolan et al., 2012; Wulff et al., 2021) and dance interventions (Branson Dame et al., 2024). Research has demonstrated the effectiveness of these protocols in promoting maternal wellbeing during pregnancy. For instance, relaxation techniques such as through yoga, meditation or muscle relaxation have been positively associated with reduced anxiety and depressive symptoms reduction (for a review see Abera et al., 2024). Similarly, music listening, as well as singing, has been shown to reduce maternal stress, anxiety, depression and blood pressure (e.g., Corbijn Van Willenswaard et al., 2017; Wulff et al., 2021; Baltacı and Başer, 2022; see review in Maul et al., 2024).

Crucially, evidence suggests that these types of interventions also exert a positive influence on perinatal outcomes. Studies have shown that music listening during pregnancy produces long-term plastic effects on the developing brain, enhancing neural responsiveness to sounds (Partanen et al., 2013) and speech stimuli periodicity (Arenillas-Alcón et al., 2023). In a similar vein, other studies showed that prenatal exposure to music impacts neonatal neurobehavioural assessments (e.g., Arya et al., 2012; Rochanah et al., 2020). The positive effects of prenatal interventions have been also observed in foetal movements (Rochanah et al., 2020), gestational age at birth (Rajeswari and Sanjeeva Reddy, 2020), Apgar scores (e.g., Chuntharapat et al., 2008; Beevi et al., 2017; García González et al., 2017), birth weight (e.g., Beevi et al., 2017; García González et al., 2017; Rajeswari and Sanjeeva Reddy, 2020), and birth length (Ahmadi et al., 2019). However, while the evidence for the positive impact of these types of interventions on maternal wellbeing is strong, the evidence for their effectiveness on perinatal outcomes is more variable and less definitive.

One aspect in particular that has been overlooked in the literature is that related to the possible specificity of the effects produced on maternal wellbeing and neonatal outcomes by prenatal programmes involving passive relaxation practices or active maternal engagement. While passive relaxation primarily promotes stress and arousal reduction (e.g., Pelletier, 2004), active engagement (e.g., physical activity) typically enhances psychophysiological activation (e.g., Goldsmith et al., 2000; Kang et al., 2018). Therefore, it is reasonable to hypothesise that prenatal interventions involving passive (i.e., relaxation-based) versus active (i.e., activity-based) protocols may affect different or act differently on specific dimensions of maternal wellbeing. With regard to maternal wellbeing, only a few studies have provided indirect evidence in this regard, showing mixed results. A recent meta-analysis of 32 studies focusing on the effects of prenatal relaxation interventions shows that passively listening to music is more effective than actively engaging in yoga or breathing exercises during music listening (Abera et al., 2024) in reducing pregnant women’s stress and anxiety levels. However, Wulff et al. (2021) obtained different results by comparing the effects of passive listening to classical music and active singing on both psychophysiological correlates of stress and bonding and self-reported measures of anxiety and depression symptoms. Although neither of the interventions affected self-reported anxiety and depression, they were both associated with increased oxytocin levels and decreased cortisol levels, with this last effect being greater for the singing group. Moreover, they found that the singing protocol produced a larger improvement in the perceived closeness to the foetus compared to the relaxation protocol.

Regarding neonatal outcomes, the evidence is also mixed. The meta-analysis by Abera and colleagues (Abera et al., 2024) shows that, although with significant heterogeneity among the included studies (i.e., *I*^2^ = 63%), prenatal relaxation interventions promote vaginal delivery, reduce labour duration, and positively impact gestational age and birth weight. However, additional studies investigating the impact of either prenatal music or singing stimulation yielded null results for these same outcomes (Arya et al., 2012; Persico et al., 2017).

**Figure 1:**
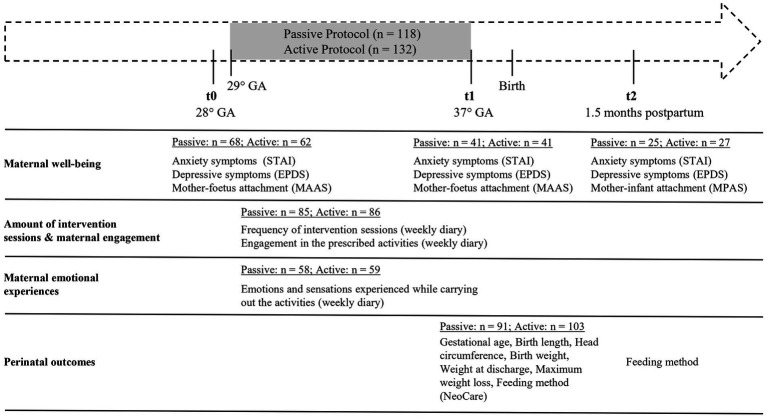
Timeline of the study and type of data with corresponding sample sizes by time of collection. GA, Gestational Age; STAI, Spielberg State–trait Anxiety Inventory; EPDS, Edinburgh Postnatal Depression Scale; MAAS, Maternal Antenatal Attachment Scale; MPAS, Maternal Postnatal Attachment Scale.

Given the limited and inconsistent evidence available in the literature regarding the unique and shared impact of passive and active prenatal interventions, the present study aims to explore and compare the effects of two novel prenatal intervention protocols differing in terms of active versus passive engagement on maternal wellbeing (i.e., emotional experience, depression, anxiety level and bonding), and perinatal outcomes. To create this distinction, we combined a series of daily-life activities commonly performed by mothers during pregnancy (e.g., Costa et al., 2003) that, taken together, would imply a more active or passive maternal engagement. We intended to maximise the ecological validity of the interventions, with the final aim of potentially offering alternatives that may accommodate individual preferences towards active and reflective activities, maximising the effectiveness of the interventions and promoting their universal application. One protocol emphasised relaxation (Passive Protocol), while the other involved the mother’s active engagement in various motor activities (i.e., tapping, bouncing) during music listening (Active Protocol). Two groups of pregnant women were recruited and firstly assessed at 28 weeks gestation (t0), and were followed longitudinally after the protocol (t1) and up to 1.5 months postpartum (t2) (see Figure 1). The study had four main goals: (a) to compare the impact of the Passive versus Active prenatal protocol in promoting maternal wellbeing in terms of reduction of anxiety and depressive symptoms and positive changes in mother-foetus/infant bonding; (b) to compare the emotional experience elicited in the participants by the two types of interventions; (c) to compare the impact of the two prenatal intervention protocols on perinatal outcomes; (d) to explore whether the impacts of the protocols on maternal wellbeing, emotional experience, and/or perinatal outcomes were modulated by the number of intervention sessions and/or amount of maternal engagement with the prescribed activities.

In general, the overarching hypothesis was that while relaxing activities may facilitate lowering of arousal, encourage self-awareness and provide an opportunity for deeper reflection, rhythmic activities involving the foetus (tapping) may promote precursors of parental behaviours by stimulating interaction. Specifically, based on existing evidence, we hypothesised that both intervention groups would experience a decrease in (state) anxiety and depression symptoms over time, with the Passive Protocol group demonstrating a greater reduction compared to the Active Protocol group. Moreover, we expected to observe an increase in bonding and attachment levels in both groups, with a more pronounced effect expected for the Active Protocol group. We predicted that the two protocols would differentially impact participants’ emotional experiences throughout the last trimester of gestation, reflecting the passive versus active nature of the prescribed activities. Finally, we hypothesised that adherence to the intervention protocol and/or the amount of maternal engagement with the prescribed activities would significantly modulate the observed effects. Regarding perinatal outcomes, given the scarcity of consistent evidence in the literature, we approached this study with an exploratory mindset, without making any specific predictions.

## Method

The study was designed as a longitudinal interventional study in which pregnant women were followed from 28 weeks of gestation up to 1.5 months postpartum to investigate the impact of two prenatal intervention programmes on perinatal maternal wellbeing (i.e., anxiety and depression symptoms, mother-foetus/infant bonding) and perinatal outcomes (mode of delivery, gestational age, birth weight, length and cranial circumference). Women with uncomplicated pregnancies were randomly assigned to one of two intervention groups: the Passive Protocol group, who was instructed to passively listen to soothing nature sounds while seated or lying down, or the Active Protocol group, who was instructed to actively listen to pop music while performing a range of activities conveying multisensory stimulation to the foetus. Different measures of maternal wellbeing were collected at three time points: t0 (28 weeks of gestation, before intervention), t1 (37 weeks of gestation, post intervention), t2 (1.5 months postnatal, follow-up). During the intervention (i.e., between t0 and t1; see Figure 1), pregnant women were asked to fill-in a weekly diary including specific questions aimed at measuring the number of intervention sessions performed during the week, the extent of the participant’s engagement with the prescribed activities, and the feelings and emotions experienced. Information about perinatal outcomes were collected at t1 for all term-newborns. The study was approved by the Milano Area 2 Ethical Committee, Fondazione IRCCS Ca′ Granda Ospedale Maggiore Policlinico (ID: 694; Approval N. 952_2021), and participants completed an informed consent before the study began.

### Participants

Participants were recruited on a voluntary basis from childbirth education classes offered at the Ospedale Maggiore Policlinico of Milan. Invitations to participate were sent via email by the hospital, and interested women completed an online survey on Qualtrics^1^ to provide contact details. Italian speaking pregnant women over 18 years were considered eligible. Following an informative online meeting conducted by a researcher, the enrolled participants were randomly assigned by another researcher, who was not involved in the meeting, to one of the two groups: Passive Protocol or Active Protocol. Following group assignment, participants received the informed consent form via email; once consent was obtained, they were supplied with the materials required for their assigned activities. The final sample included 250 participants (Passive Protocol group, *N* = 118; Active Protocol group, *N* = 132) who completed the intervention protocols they were assigned to, but sample size varied over the different assessment sessions due to factors such as participant availability and accessibility over time (see Figure 1). Pregnant women were 20–46-year-old (*M* = 35.69, *SD* = 4.81) at the time of recruitment. An additional 52 women participated in some or all assessment sessions but were excluded from the final sample because of complications during pregnancy (*N* = 8) or childbirth (*N* = 3), preterm birth (*N* = 9), newborn’s health issues (*N* = 6), expressed decision to interrupt the intervention protocol and withdraw from the study (*N* = 26). Sample size was initially estimated based on a prior study with a similar study design investigating the effects of prenatal interventions on maternal mental health (Wulff et al., 2021). However, as it has been noted that most prior research on prenatal interventions was primarily powered to examine impacts on maternal mental health while being underpowered to detect effects on neonatal outcomes (see review in Abera et al., 2024), an additional power analysis was conducted to ensure adequate sample size for the detection of potential effects on neonatal outcomes. The analysis was conducted using Gpower software (version 3.1.9.4) to determine the required sample size for detecting a difference between two independent means (two groups) with a medium effect size (*d* = 0.5 an alpha error probability of 0.05), and a statistical power of 0.80. The analysis indicated that a total sample size of 102 participants (51 per group) would be required. To account for potential participant attrition, we recruited a significantly larger sample of *N* = 302. Sample characteristics at the first time of assessment (t0) are summarised in Table 1. All characteristics were comparable in the two groups (all *ps* > 0.24).

**Table 1:** Maternal characteristics at t0, by study group.

Sample characteristic	Passive protocol group (*n* = 118)		Active protocol group (*n* = 132)	
Demographics	Mean	SD	Mean	SD
Mother’s age (years)	36	4.35	35.5	5.20
Education*	5.52	1.27	5.28	1.42
Employment status**	3.97	0.95	4.14	0.82
SES***	9.25	2.09	9.36	2.06
Marital status	n	%	n	%
In a stable relationship	116	98.3	129	97.73
Years of relationship	Mean	SD	Mean	SD
	9.11	4.62	8.41	5.20
Nutrition	n	%	n	%
Vegetarian	2	1.69	6	4.55
Vegan	4	3.39	0	0
Obstetric	n	%	n	%
Primiparous	83	70.3	94	71.2
MAP cases	23	19.5	24	18.2
GA at recruitment	Mean	SD	Mean	SD
	23.5	4.46	23.7	5.45

GA, Gestational Age; MAP, Medically Assisted Procreation.

*Maternal education was scored on a 7-point scale: (1) primary school; (2) junior high school; (3) vocational degree; (4) high school; (5) bachelor’s degree; (6) five-year master’s degree; (7) doctoral degree. **Maternal employment was scored on a 5-point scale: (1) occupations with minimal skills, no qualifications, or advanced training; (2) occupations that require basic training or role-specific skills; (3) occupations requiring an intermediate technical qualification; (4) occupations requiring a moderate level of qualification and specialised training; (5) highly qualified occupations. ***SES (Socio-Economic-Status) was calculated as the sum of education and employment scores.

### Protocol pipeline

Pregnant women, randomly assigned either to the Passive Protocol group or the Active Protocol group, were instructed to engage in the intervention activities starting from the 29th week of gestation, with a recommended frequency of at least 4 times per week, each lasting at least 15 min. They were provided with written instructions and a diary to record their activities and experiences. Along with background information on foetal development and research references, the diary contained a series of questions to be completed each week. These questions pertained to the frequency of intervention sessions carried out in the week, the specific musical tracks selected during each session, the level of engagement with the prescribed activities, and the emotional responses experienced during the sessions (see Supplementary Figure S1 in the Supplementary Information S1). During the intervention period, expectant mothers were offered the opportunity to participate in online meetings to express doubts, ask questions, and share their experiences with other participants.

#### Passive prenatal intervention protocol

The Passive Protocol consisted of listening to tracks to be selected from a playlist of relaxing sounds (i.e., nature sounds or white noises; see Supplementary Information S2 for a description of soundtrack selection) through speakers at a regular volume for at least 15 min, 4 times per week. While listening to the tracks, women were instructed to lie down or sit, and relax as much as possible. The use of relaxing sounds instead of relaxing music in the passive protocol allowed to enhance the contrast between the two interventions and minimise the potential confounding effects associated with incorporating music in both protocols, as observed in some previous studies (e.g., 26).

#### Active prenatal intervention protocol

The active protocol involved performing activities designed to provide multisensory stimulation to the foetus. Participants were requested to listen to songs (approximately 75–85 decibel Sound Pressure Level), hum along with the melodies, rhythmically tap their abdomen and gently bounce around in sync with the rhythm of the music. Participants were instructed to engage in these activities at least 4 times per week, for at least 15 min per session. While a pre-selection of 20 songs was provided through YouTube or Spotify playlists (see Supplementary Information S2 for a description of songs selection), mothers were allowed to expand this selection by adding their own preferred songs if desired.

## Measures

### Maternal wellbeing

#### Anxiety symptoms—Spielberg State–trait Anxiety Inventory—STAI

The Spielberg State–trait Anxiety Inventory (STAI; Spielberger et al., 1971) is a widely used tool for assessing maternal anxiety. The questionnaire is divided into State and Trait scales, each composed of 20 items on a 4-point Likert scale ranging from 1 (Not at all) to 4 (Very much). State-STAI measures situational and transient anxiety, reflecting the temporary emotional response to specific stressors or events. Trait-STAI, on the other hand, assesses the stable individual predisposition to experience anxiety across different contexts and situations. Total scores for each subscale range from 20 to 80, with higher scores indicating higher levels of anxiety.

#### Depression symptoms—Edinburgh Postnatal Depression Scale—EPDS

To assess levels of depression, the Edinburgh Postnatal Depression Scale (EDPS; Cox et al., 1987) was administered. It is composed of 10 questions on a 4-point Likert scale that assess the depressive symptoms experienced by the participant in the past 7 days. Scores range from 0 to 30, with higher scores indicating higher depression levels. The questionnaire can be used both during gestation and after delivery (Cox et al., 1996).

#### Maternal Antenatal Attachment Scale—MAAS

The Maternal Antenatal Attachment Scale (*MAAS*; Condon, 1993) is a 19-items self-report questionnaire designed to assess the quality of the mother’s attachment to the foetus, rated on a 5-point Likert scale. The questions, rated on a 5-point Likert scale, are grouped in two categories, assessing two different dimensions: Intensity of preoccupation (*MAAS-I*; 8 questions), evaluating the amount of time spent by mothers thinking, talking, and dreaming about their unborn baby, and Attachment quality (*MAAS-Q*; 11 questions), referring to the mothers’ closeness, tenderness, and positive feelings towards the foetus. The global attachment score ranges from 19 to 95 with higher scores indicating a higher level of attachment to the foetus.

#### Maternal Postpartum Attachment Scale—MPAS

The Maternal Postpartum Attachment Scale (MPAS; Condon and Corkindale, 1998; Scopesi et al., 2004) consists of 19 items with response options on a 2-, 3-, 4-, and 5-point scale, depending on the item. Condon and Corkindale (1998) identified three dimensions: pleasure in interaction with the infant (*MPAS-P*; 5 items), absence of hostility towards the infant (*MPAS-A*; 5 items), and quality of mother–infant attachment (*MPAS-Q*; 9 items). The total score ranges from 19 to 95, with higher scores indicating greater maternal postnatal attachment to the baby.

### Participants’ emotional experiences

Participants’ Emotional experiences during the prescribed activities were assessed through a single open-ended question in the weekly diary: “*What emotions and sensations did you experience while carrying out the activity?*.” At the end of the study, participants provided feedback on their experiences with the intervention and any difficulties encountered. Answers to the open-ended question were subjected to thematic analysis, which led to identification of four major themes: Positive emotions, Mother-foetus relation, Daily coping, and Negative emotions (see Supplementary Information S3 for a detailed description of the method adopted).

### Perinatal outcomes

Data on perinatal outcomes were collected from computerised medical records (Neocare i&t Informatica e Tecnologia Srl, Italy), and are listed in Table 2. They included delivery mode, gestational age, birth weight and length, head circumference, data collected at discharge like feeding method, weight, maximum weight loss during birth hospitalisation, and feeding method at 1.5 months.

**Table 2:** Neonatal characteristics at birth and 1.5 months, by study group.

Sample characteristic	Passive protocol group (*n* = 118)		Active protocol group (*n* = 132)	
Delivery	n	%	n	%
Vaginal	58	49.15	61	46.21
Newborn	Mean	SD	Mean	SD
Gestational age (weeks)	39.51	1.19	39.31	1.43
Birth length (cm)	49.66	1.76	49.57	1.80
Birth length %ile	46.76	28.86	48.10	27.07
Head circumference (cm)	34.32	1.03	34.52	1.22
Head circumference %ile	52.22	26.99	58.05	27.07
Birth weight (gr)	3,196.96	367.75	3,222.82	404.28
Birth weight %ile	40.56	26.64	46.35	28.56
Gender (female)	n	%	n	%
	57	48.31	62	46.97
At discharge	Mean	SD	Mean	SD
Weight (g)	2,962.25	318.91	3,001.17	375.07
Max % Weight loss	8	0.02	8.9	0.08
Breastfeeding	n	%	n	%
	60	50.58	61	46.21
Breastfeeding at 1.5 months	76	64.41	80	60.61

%ile, percentile.

### Number of intervention sessions and maternal engagement

To monitor adherence to the intervention protocol and maternal engagement, participants were asked to complete a weekly diary reporting:

- *Frequency of interventions sessions*: the number of intervention sessions conducted each week and the specific soundtracks used from the provided playlist (allowing for the addition of other songs for the Active Protocol group).
- *Engagement with the prescribed activities*: how much they relaxed on a 4-point Likert scale (Passive Protocol group) or how much they hummed, tapped, and bounced (Active Protocol group) during the intervention sessions conducted each week, as rated on three separate 5-point Likert scales. For the Active Protocol group, a total Active Engagement score was calculated by summing the scores (ranging from 1 to 5) across the three questions regarding humming, tapping, and bouncing. These weekly scores were then averaged across all weeks of the intervention. For the Passive Protocol group, a total Relaxation Engagement score was calculated by averaging the weekly scores obtained on the 4-point Likert scale assessing the level of relaxation during the sessions. Supplementary Figure S1 in the Supplementary Information S1 shows the pages of the diaries that mothers were required to fill.

### Statistical analysis

Statistical analyses were conducted using the R statistical software (R Core Team, v. 2021). To test the effects of the two prenatal intervention protocols on maternal wellbeing, three separate linear mixed models were performed for each maternal outcome variable: anxiety symptoms, depression symptoms, and mother-foetus/infant bonding. The main statistical models tested the effect of group (Passive, Active), Timepoint (t0, t1, t2), and their interaction as fixed factors, and random intercept for participants. For the mother-foetus/infant bonding outcome, we tested Group × Timepoint (t0, t1) effects on the MAAS subscales (intensity of preoccupation, attachment quality), and we assessed the effect of group on the MPAS subscales (pleasure in interaction, absence of hostility towards the infant, quality of mother-infant attachment) at t2.

To compare the emotional experiences of participants in the two groups, we performed a MANOVA with group (passive, active) as independent variable and the frequency of themes identified through thematic analysis of the weekly diary entries as dependent variables.

To assess the impact of the two prenatal intervention protocols on perinatal outcomes, we conducted a MANOVA with group (passive, active) as independent variable and gestational age, birth weight, length, cranial circumference, birth at discharge, and maximum weight loss as dependent variables. Additionally, Chi-squared tests were performed to explore group differences in delivery mode (vaginal, caesarean) and feeding method (breastfeeding, formula, mixed) at discharge and 1.5 months. To account for possible unmeasured confounders, a sensitivity analysis was performed for each of the perinatal outcomes. To this end, we calculated the *E-value* for each of the perinatal outcomes, following guidelines in Vander Weele and Ding (2017). The *E-value* is the minimum strength of association, in terms of risk ratio, that an unmeasured confounder would need to have with both the intervention and the outcome to completely account for a specific intervention-outcome association, conditional on the measured covariates. *E-values* < 1.5 indicates that the result may be sensitive to unmeasured confounders.

To assess whether there were differences in intervention adherence and maternal engagement between groups, an independent sample *t*-test was conducted to compare the mean number of intervention sessions completed between the Passive Protocol and the Active Protocol groups. To examine the impact of the number of intervention sessions and amount of maternal engagement on maternal wellbeing at t1, a series of linear regression analyses were performed for each maternal outcome variable (anxiety, depression, and mother-foetus/infant bonding), separately for each group. In these models, Δ scores (scores at T0-scores at T1 for STAI and EPDS; scores at T1-scores at T0 for MAAS) were used as the dependent variable, while the frequency of intervention sessions, the Active Engagement score (for the Active Protocol group) or the Relaxation Engagement score (for the Passive Protocol group) were included as independent variables. Linear regressions with the themes identified through thematic analysis as dependent variables and the frequency of intervention sessions and maternal engagement as independent variables were also run separately for each group. Lastly, within each group, we performed linear regression analyses to test the impact of the number of intervention sessions and maternal engagement on perinatal outcomes. Additionally, we performed binomial and multinomial logistic regressions to investigate the effects of the number of intervention sessions and maternal engagement on delivery mode (vaginal, caesarean) and feeding method (breastfeeding, formula, mixed).

## Results

### Comparison of maternal wellbeing across intervention protocols

**Figure 2:**
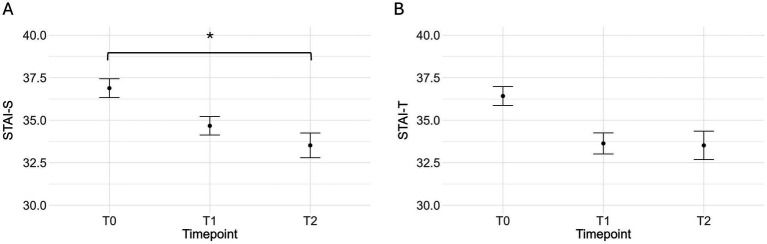
Mean (and standard error) state- **(A)** and trait- **(B)** anxiety scores at the three different timepoints across the two protocol groups. *p < 0.05.

Linear mixed models on State-Anxiety scores revealed a significant main effect of Timepoint *F*(2,144.19) = 4.65, *p* = 0.011. Post-hoc comparisons indicated a significant decrease in state anxiety levels from t0 (*M* = 36.9, *SE* = 0.82) to t2 (*M* = 33, *SE* = 1.24), *t*(185) = 2.92, *p* = 0.011 across both groups (Figure 2). Polynomial contrast tests confirmed a linear trend over time, *t*(185) = 2.92, *p* = 0.004, with no evidence of a quadratic trend (*p* = 0.89). No significant Group or Group × Timepoint effects were found, (all *ps* > 0.08). For Trait-Anxiety, no significant differences (all *ps* > 0.06) were found for Group or Timepoint effects.

**Figure 3:**
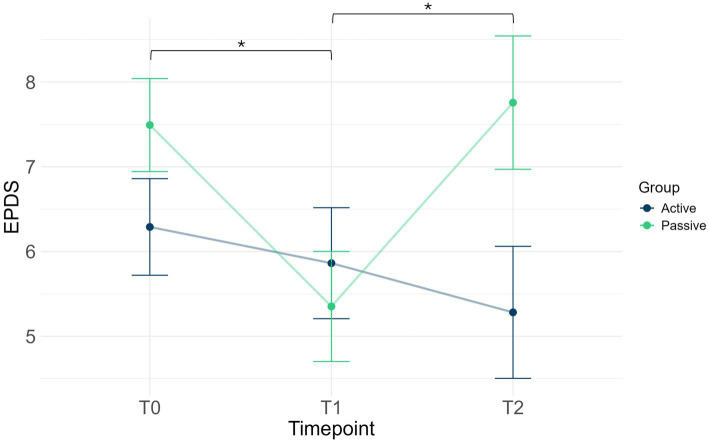
Mean (and standard error) depression scores at the three different timepoints in the active protocol (blue line) and passive protocol (green line) groups. *p < 0.05.

Linear mixed models on Depression scores showed a main effect of Timepoint, *F*(2,133.94) = 4.5, *p* = 0.01, which was qualified by a significant Group × Timepoint interaction, *F*(2,133.94) = 3.62, *p* = 0.03. Tukey-corrected *post hoc* comparisons revealed that, while the Passive Protocol group showed a significant decrease in depression levels from t0 (*M* = 7.49, *SE* = 0.55) to t1 (*M* = 5.35, *SE* = 0.65), *t*(130) = 3.55, *p* = 0.007, followed by a marginal increase from t1 to t2 (*M* = 7.76, *SE* = 0.79), *t*(146) = 2.89, *p* = 0.05, depression levels remained stable across Timepoints for the Active Protocol group (all *ps* > 0.8) (Figure 3).

**Figure 4:**
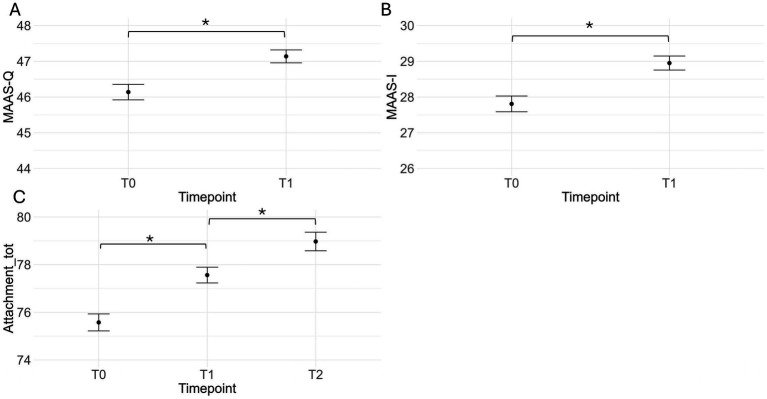
Panels **(A,B)** show the mean (and standard error) scores for quality of attachment **(A)** and intensity of preoccupation **(B)** from the MAAS at timepoints t0 and t1 across the two protocol groups. Panel **(C)** depicts mean attachment scores across the three timepoints as measured by the MAAS and MPAS. *p < 0.05.

Linear mixed models on mother-foetus/infant bonding showed a main effect of Timepoint, *F*(2, 137.48) = 11.8, *p* < 0.001. Tukey-corrected post hoc comparisons indicated a significant increase in mother-foetus/infant attachment from t0 (*M* = 75.4, *SE* = 0.49) to t1 (*M* = 76.8, *SE* = 0.58), *t*(127) = 2.5, *p* = 0.04, and from t1 to t2 (*M* = 78.8, *SE* = 0.71), *t*(158) = 2.55, *p* = 0.03, with no significant group differences. Further analyses for the MAAS subscales revealed significant increases in both Quality of attachment, *F*(1, 77.86) = 8.05, *p* = 0.006, and Intensity of preoccupation *F*(1, 84.41) = 6.59, *p* = 0.012, from t0 (MAAS-Q: *M* = 46, *SE* = 0.2; MAAS-I: *M* = 27.7, *SE* = 0.29) to t1 (MAAS-Q: *M* = 46.7, *SE* = 0.32; MAAS-I: *M* = 28.5, *SE* = 0.34). No significant group differences were found at t2 in the scores obtained in the MPAS-P, MPAS-A and MPAS-Q subscales (all *ps* > 0.31). Figure 4 graphically depicts the results of the linear mixed model analyses on maternal wellbeing.

### Comparison of maternal emotional experience across intervention protocols

**Figure 5:**
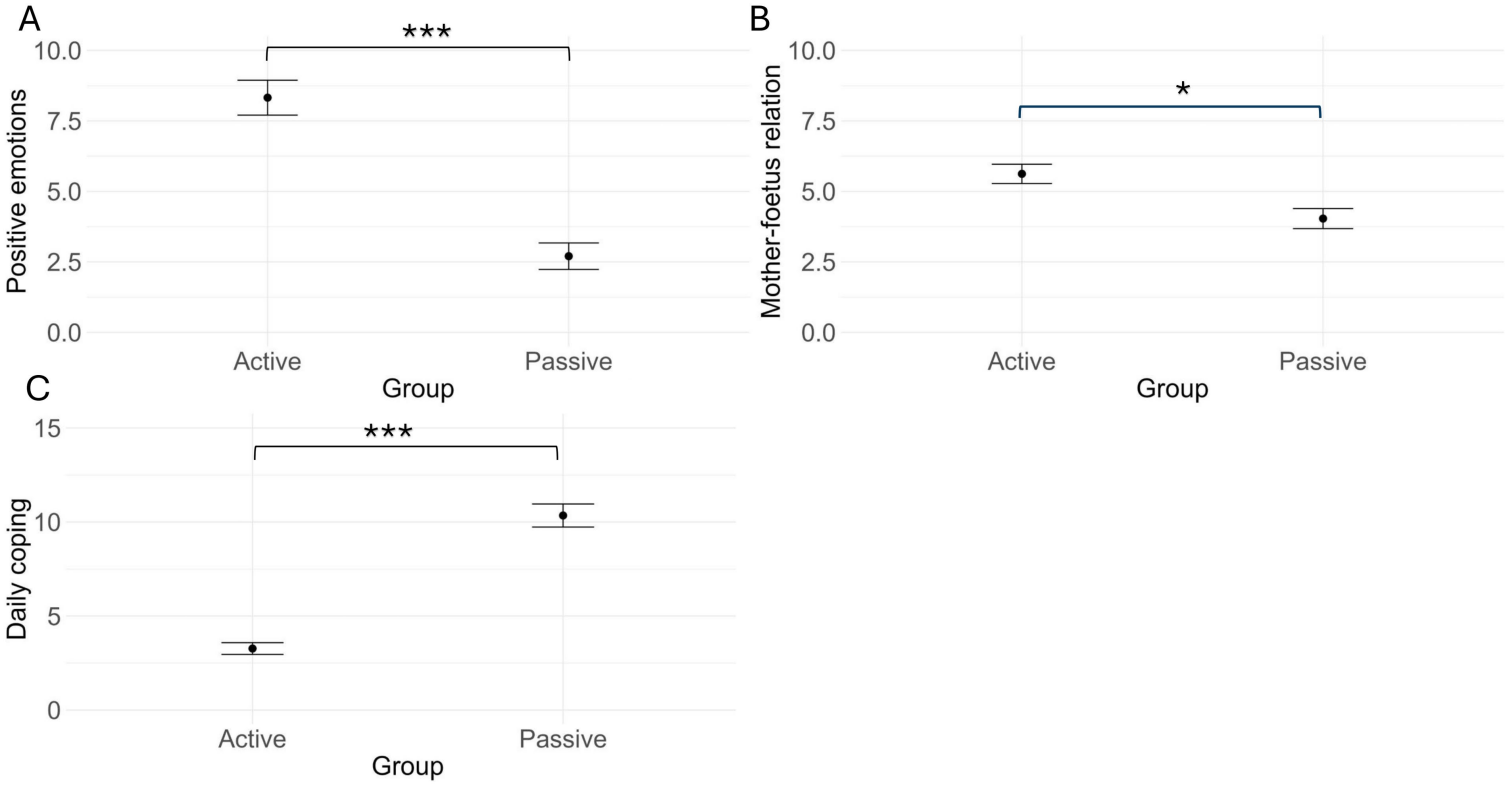
Mean and standard error of the ‘positive emotion’ **(A)**, ‘mother-foetus relation’ **(B)**, and ‘daily coping’ **(C)** theme frequencies for the active protocol and the passive protocol groups. *p < 0.05; ***p < 0.001.

The MANOVA performed on the themes identified through thematic analysis revealed significant group differences in the frequency of specific themes, *V*(1) = 0.55, *F*(4,105) = 31.46, *p* < 0.001. The Active Protocol group reported significantly higher frequency of ‘Positive emotions’ (*M* = 8.3, *SE* = 0.62) and ‘Mother-foetus relation’ (*M* = 5.62, *SE* = 0.36) themes in their diaries entries compared to the Passive Protocol group (positive emotion: *M* = 2.7, *SE* = 0.47, *F*(1,108) = 19.29, *p* < 0.001; mother-foetus relation: *M* = 4.04, *SE* = 0.34, *F*(1,108) = 4.62, *p* = 0.03). Conversely, the frequency of ‘Daily coping’ themes was significantly higher in the Passive Protocol group (*M* = 10.3, *SE* = 0.61) compared to the Active Protocol group (*M* = 3.27, *SE* = 0.31), *F*(1,108) = 50.66, *p* < 0.001. No significant group differences were found in the frequency of ‘Negative emotion’ themes, *F*(1,108) = 3.38, *p* = 0.07 (Figure 5).

### Comparison of perinatal outcomes across intervention protocols

Table 2 summarises neonatal characteristics at birth.

The MANOVA performed on perinatal outcomes revealed no differences between the Active and Passive Protocol groups in terms of gestational age, birth weight or length, head circumference, weight at discharge and maximum weight loss, *V*(1) = 0.04, *F*(7,185) = 1.003, *p* = 0.43. Similarly, no significant group differences were observed in delivery mode or feeding method at discharge and 1.5 months (all *ps* > 0.27). Results of the sensitivity analyses revealed that while birth weight (*E-valu*e = 1.5) and length percentile (*E-value* = 1.51) exhibited moderate sensitivity to unmeasured confounders, delivery mode (*E-value* = 1.1) was highly sensitive to unmeasured confounders.

### Impact of the amount of intervention sessions and maternal engagement on maternal wellbeing and perinatal outcomes

The mean number of intervention sessions conducted was 133.01 for the Active Protocol group (*SD* = 55.3) and 132.61 (*SD* = 54.9) for the Passive Protocol group. An independent-sample *t*-test revealed no significant differences between groups, *t*(167.95) = 0.05, *p* = 0.96.

**Figure 6:**
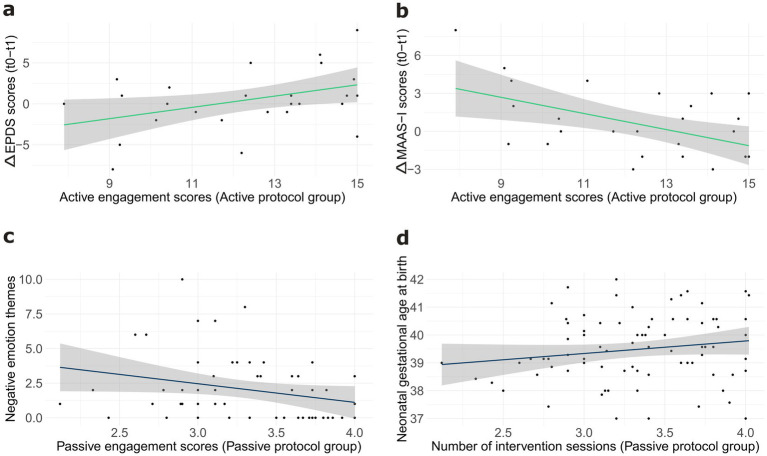
Panel **(a)** depicts the association between the active engagement scores and change in depression scores between t0 and t1 in the active protocol group. Panel **(b)** illustrates the association between the active engagement scores and change in Intensity of Preoccupation scores between t0 and t1 in the active protocol group. Panel **(c)** depicts the association between relaxation engagement scores and the frequency of the ‘negative emotion’ themes reported by mothers in the passive protocol group. Panel **(d)** shows the association between the number of intervention sessions and neonatal gestational age at birth in the passive protocol group. Grey areas display confidence intervals.

Linear regressions analyses revealed no significant associations between the number of intervention sessions or the amount of maternal engagement (Active Engagement scores for the Active Protocol group, and Relaxation Engagement scores for the Passive Protocol group) and changes (Δ anxiety scores) in State-Anxiety (all *ps* > 0.4) or Trait-Anxiety (all *ps* > 0.09) scores between t0 and t1 in either group. For depression scores, results of linear regression showed that, for the Active Protocol group, the difference between depression levels (Δ depression scores) at t0 and t1 was significantly predicted by the Active Engagement scores, *b* = 0.69, *t*(26) = 2.26, *p* = 0.03, suggesting that greater engagement with active singing/humming, tapping, and dancing was associated with a larger decrease in depressive symptoms (Figure 6). No other significant associations were found between intervention adherence or engagement and maternal wellbeing outcomes (all *ps* > 0.07).

For mother-foetus bonding, regression analyses revealed a significant positive association between Active Engagement scores and change in Intensity of Preoccupation (MAAS-I) scores in the Active Protocol group, *b* = 0.64, *t*(25) = 2.93, *p* = 0.007. Specifically, higher levels of active engagement were associated with smaller increases in MAAS-I scores from t0 to t1 (Figure 6), suggesting that greater engagement with the prescribed activities may have mitigated increases in the Intensity of preoccupation with the foetus. No significant associations were found between the number of intervention sessions or engagement scores and other maternal wellbeing outcomes (all *ps* > 0.17).

Linear regression analyses for emotional experiences revealed a marginally significant negative association between Relaxation Engagement scores and the frequency of ‘Negative emotion’ themes in the Passive Protocol group, *b* = −1.3457, *t*(52) = 1.96, *p* = 0.05 (Figure 6), suggesting that lower levels of relaxation engagement during the passive protocol were associated with a larger increase in negative emotions reported by mothers.

Linear regression analyses for perinatal outcomes showed a significant positive association between the number of intervention sessions and gestational age at birth, *b* = 0.007, *t*(81) = 3.24, *p* = 0.002 for the Passive Protocol group (Figure 6). No other significant associations were found between intervention adherence (number of intervention sessions) or maternal engagement (Active Engagement, Relaxation Engagement) and any of the perinatal outcomes measured (all *ps* > 0.1).

## Discussion

The primary aim of this study was to explore and compare the impact of two novel prenatal intervention programmes on maternal wellbeing and neonatal outcomes: a passive programme adopting a relaxation-based protocol, and an active programme involving the mother’s active engagement with various activities designed to enhance foetal multisensory experience. Expectant mothers at 28 weeks of gestation were randomly assigned to one of the two intervention groups. A mixed-method approach, combining quantitative and qualitative measures, was employed to longitudinally assess the impact of the intervention protocols on maternal wellbeing and neonatal outcomes. A novel aspect of this study was the inclusion of a follow-up assessment a few months postpartum to examine the long-term impact of the prenatal interventions on maternal wellbeing outcomes, which has been largely overlooked in previous research.

Both quantitative and qualitative measures indicated that the two prenatal intervention protocols had a positive impact on maternal mental health, exhibiting both similarities and differences. In both groups, state-anxiety levels decreased from t0 to t2, with a linear decrement across timepoints. This finding replicates and extends previous evidence on the effectiveness of prenatal interventions in reducing state-anxiety symptoms during pregnancy (e.g., Bastani et al., 2005; Sanfilippo et al., 2021), indicating that these beneficial effects persist into the postpartum period. In contrast to the consistent decrease in anxiety levels observed across groups, depression symptoms exhibited different trajectories. While the Passive Protocol group showed a significant decrease from t0 to t1 followed by an increase from t1 to t2, depression levels remained relatively stable across timepoints in the Active Protocol group. This suggests that the Passive Protocol may be particularly effective in supporting emotional regulation during pregnancy. Pregnancy is a period of profound physiological and psychological changes, and emotional regulation is crucial in managing stress, promoting maternal-foetal bonding, and preparing for labour (Penner and Rutherford, 2022). Relaxation practices in this regard may be particularly beneficial during this time by reducing cortisol levels and anxiety and promoting calmness (Ventura et al., 2012). However, these benefits may not extend to the postpartum period, which introduces new physiological, physical and psychological challenges. Potential stressors like postpartum stress, newborn care demands, economic challenges, sleep deprivation, and hormonal changes, may have interfered with the protocol’s benefits, suggesting the need for different types of intervention to effectively address postpartum mental health needs, such as social support (Bedaso et al., 2021; Takács et al., 2021). Besides, the observed increase in depressive symptoms during the postpartum period may be also explained by the abrupt discontinuation of the relaxation protocol after childbirth. Given that evidence suggests the effectiveness of postnatal relaxation techniques in reducing postpartum depressive symptoms (Gökşin and Ayaz-Alkaya, 2020), it is plausible to hypothesise that, if the mothers had continued their engagement with relaxation practices prescribed by the protocol beyond pregnancy, this may have mitigated the observed increase in depression scores in the postpartum. However, it is important to note that the high participant drop-out rate at t2 significantly impacted our final sample size, possibly resulting in underpowered analyses at this timepoint.

The finding that the reduction in depressive symptoms from t0 and t1 was observed primarily in the Passive Protocol group is consistent with the qualitative data derived from the weekly diaries. While participants in both groups experienced negative emotions (e.g., anxiety, sadness) during the intervention period, women in the Passive Protocol group, who reported higher levels of relaxation engagement, also reported fewer ‘Negative emotions’ themes. On the other hand, there was no significant association between active engagement and the frequency of negative emotions reported by mothers in the Active Protocol group. Overall, these findings align with those from a recent meta-analysis (Abera et al., 2024) that showed a stronger association between passive listening to relaxing music and reductions in depressive symptoms compared to more active forms of prenatal relaxation interventions, such as yoga and meditation.

While the overall group-level analysis did not reveal a significant timepoint effect on depression scores in the Active Protocol group, linear regression analyses on the qualitative data collected through the weekly diaries revealed a more nuanced picture. Specifically, the level of active engagement with the prescribed activities reported by the mothers predicted the reduction in depressive symptoms from t0 to t1, with higher levels of engagement being associated with greater reduction. This suggests that the effectiveness of the Active Protocol in reducing depressive symptoms may be contingent upon participants’ engagement. High levels of engagement with the prescribed activities (humming, tapping, and bouncing during music listening) may have enhanced enjoyment and facilitate the emergence of positive emotions, enhancing the positive impact of the intervention on mood.

Regarding mother-foetus/infant bonding, both protocol groups showed a significant increase in attachment level from t0 to t1, as evidenced by increases in both Intensity of Preoccupation and Attachment Quality subscales of the MAAS. Of note, global attachment scores show that such an increase extends into the postnatal period (t2). This finding is consistent with previous research by Wulff et al. (2021), which showed increases in attachment scores and oxytocin levels following both relaxing and singing-based prenatal interventions. The lack of significant group differences in attachment scores in our study may be attributed to the shared emphasis placed on the motivation for prenatal interventions in promoting maternal and foetal/infant wellbeing during the recruitment phase of the study. This focus might have fostered participants’ engagement in supporting foetal development and wellbeing through the prescribed activities, inherently promoting a sense of connection with the foetus/infant, regardless of the specific intervention protocol. This interpretation aligns with the evidence that singing infant-directed songs (i.e., lullabies) has a beneficial effect on maternal–infant bonding, especially after birth (e.g., Persico et al., 2017).

Once again, while the overall group-level analysis did not reveal significant group differences in the way attachment level changed over time, further analyses of the qualitative data collected through the diaries provided a richer understanding of the participants’ experiences. While bonding themes emerged in both groups, the frequency of the ‘Mother-foetus relation’ theme was significantly higher in the Active Protocol group than in the Passive group. This finding suggests that the active engagement with the singing and/or physical activities within the Active Protocol may have fostered a sense of closeness to the foetus. Recent studies provided evidence in this direction, supporting the effectiveness of active singing in particular. For instance, Fancourt and Perkins (2017) reported that singing interventions, but not passively listening to music, produced a positive impact on mother-infant attachment during the postnatal period. Similarly, Wulff et al. (2021) found that a singing-based intervention produced larger improvements in perceived closeness to the baby compared to a relaxation-based protocol. A possible reason for these effects lies in the expressive and communicative nature of singing and dancing, which may foster maternal sense of connection to the foetus. Additionally, maternal tactile stimulation may have critically contributed to bonding. Affective touch, which activates C-tactile afferents—specialised nerve fibres responsive to gentle and caressing touch—and stimulates the insular cortex, a brain area linked to emotional processing and social bonding, facilitates emotional regulation and attachment formation (Duhn, 2010; La Rosa et al., 2024). Another way maternal gentle tapping may have worked is through interoception. The insular cortex is indeed also linked to interoceptive awareness, potentially enhancing the mother’s ability to perceive her unborn baby’s body, which, in turn, would influence her emotional responsiveness (Montirosso and McGlone, 2020). Therefore, it is possible that the prescribed gentle tapping performed by the mothers in the Active group elicited positive emotions and strengthened the affective connection with the foetus.

An interesting finding of our study is that, in the Active Protocol group, greater active engagement was associated with a smaller increase in the Intensity of Preoccupation at t1. Intensity of Preoccupation measures the degree to which a mother worries about and thinks about her unborn baby, which is associated with greater receptivity and seeking of signals (e.g., foetal movements) from the foetus (e.g., Branjerdporn et al., 2021). Given that previous research has shown a strong link between musical and dancing activities and enhanced interoceptive abilities (e.g., Christensen et al., 2018), it is plausible that engaging in the singing and motor activities within the Active Protocol group may have improved interoceptive awareness of internal bodily signals in these participants. This heightened awareness could have led mothers to embrace the normal physical sensations of late pregnancy, such as foetal movements and uterine contractions, responding with a sense of calm rather than worry. The role of interoceptive awareness as a potential mediating factor in the reported effects of prenatal intervention programmes should be the focus of future investigation.

Despite the observed differences in their specific effects, both the Passive and Active protocols had a positive impact on maternal wellbeing, including reduction in anxiety and depressive symptoms and enhancements in mother-foetus attachment during pregnancy. A shared mechanism underlying these positive effects of both intervention protocols may be an enhancement in maternal self-efficacy. Previous research has shown that participation in prenatal interventions can significantly increase maternal self-efficacy (Wulff et al., 2021). By engaging in these activities aimed at improving maternal and foetal wellbeing, women may experience a heightened sense of self-efficacy, leading to an enhanced feeling of being in control and increased confidence in their ability to navigate the challenges of pregnancy and childbirth. This may contribute to a reduction in anxiety and depressive symptoms, ultimately promoting positive maternal mental health outcomes.

Alternatively, it is also plausible that the Passive and Active protocols operated through distinct mechanisms. Passive relaxation activities may have primarily worked by reducing physiological arousal and promoting mental imagery and states of calm (e.g., Pelletier, 2004). In contrast, engaging in music listening and motor activities might have enhanced maternal wellbeing through increased physiologic activation and the release of endorphins associated with feelings of happiness (Goldsmith et al., 2000; Kang et al., 2018), thereby promoting mood regulation. These potential differences in underlying mechanisms are supported by our qualitative data, showing some differences in the mothers’ emotional experience. In particular, while the ‘Positive emotions’ and the ‘Daily coping’ themes emerged in the diaries of both the Passive and the Active protocol groups, the Passive Protocol was associated with a higher frequency of ‘Daily coping’ themes, while the Active Protocol was associated to a higher frequency of ‘Positive emotions’ themes.

While the precise mechanisms underlying the observed effects require further investigation, these findings offer important clinical implications. Anxiety and depressive symptoms are common during pregnancy, especially during the last trimester of gestation (e.g., Teixeira et al., 2009; Gunning et al., 2010). Finding alternative strategies to pharmacological treatments is crucial, and prenatal intervention programmes represent valid and cost-effective methods to improve the wellbeing of expectant mothers. Importantly, the possibility to offer a range of intervention options allows for personalised care, enhancing their practicality and acceptability for expectant mothers.

Consistent with previous studies on maternal health outcomes of prenatal interventions (Nwebube et al., 2017; Wulff et al., 2021), we did not screen participants for pre-intervention mental health to preserve natural variability in anxiety and depression scores and track changes over time, also given evidence of the effectiveness of prenatal intervention in depressed mothers (Han et al., 2024). However, our data indicate that individual differences in initial anxiety levels influence engagement with relaxation activities in the Passive Protocol group (see Supplementary Information S4). Demographic variables, like employment and socio-economic status, also modulate engagement with the prescribed activities in the Active Protocol group (see Supplementary Information S4), again highlighting the importance of tailoring perinatal care to individual differences when offering intervention options.

In the present study, no differences between the two intervention groups were found in terms of the perinatal outcomes considered. This result contrasts with the findings of a recent meta-analysis (Abera et al., 2024), which reported that prenatal interventions increase the rate of vaginal delivery, reduce labour duration, and positively impact gestational age and birth weight. However, that meta-analysis also showed significant heterogeneity among the included studies (i.e., *I*^2^ = 63%), and it did not include studies investigating the effects of prenatal music or singing stimulation that have yielded null results for these same outcomes (Arya et al., 2012; Persico et al., 2017). Although null results are difficult to interpret, it is possible that prenatal interventions influence perinatal outcomes indirectly, by improving maternal mental health (e.g., stress level) and promoting healthy lifestyle habits (e.g., sleep quality, dietary habits). Consistent with this, our sensitivity analysis showed that birth weight, length percentile, and delivery mode were susceptible to unmeasured confounders, thus underscoring the need for cautious interpretation of these results. Interestingly, linear regression analyses including the data from the weekly diaries revealed a positive association between the number of relaxation sessions and gestational age at birth in the Passive Protocol group, suggesting that the effectiveness of the Passive protocol may be contingent upon participants’ engagement with the prescribed relaxation activities. This finding aligns with those by Rajeswari and Sanjeeva Reddy (2020), who attributed this association to a dynamic interplay of physical, psychological, and physiological mechanisms, including reductions in muscle tension, mental calmness, and reduced blood pressure and stress hormone levels. However, it is important to note that a recent meta-analysis (Abera et al., 2024) highlighted that this finding has not been consistently replicated in the literature (e.g., Bastani et al., 2005; Ghorbannejad et al., 2022). Therefore, further research is necessary to replicate this finding, and until then, our findings should be interpreted with caution. It is also worth mentioning that all newborns in our study were delivered at term, meaning that the observed variations in gestational age remained within the normal physiological range. Furthermore, the association between relaxation sessions and gestational age, while statistically significant, showed a small effect size. Despite these limitations, our results provide valuable insights. Given the potential benefits of relaxation techniques in reducing stress and promoting physiological wellbeing, investigating their potential impact on reducing the risk of preterm birth could help identify non-pharmacological interventions that support maternal health and contribute to optimal pregnancy outcomes.

This study has a number of strengths. First, it combined a quantitative approach with a qualitative analysis of the mothers’ emotional experiences during the protocol period, an approach that is often lacking in the literature. This integrated approach allowed for a richer understanding of the mother’s psychological and emotional states, which can be limited when relying solely on pre-determined questionnaire measures. Secondly, our study utilised a novel approach to the passive relaxation protocol by incorporating a selection of natural sounds and white noises, rather than relying on music as commonly employed in previous research (e.g., Wulff et al., 2021). This novel approach enhanced the contrast between the Passive and the Active protocols, minimising potential confounds associated with the use of similar auditory stimuli in both intervention groups. Thirdly, the sample size was adequately powered to detect potential effects of the interventions on perinatal outcomes, an aspect that was frequently overlooked in the literature (see review in Abera et al., 2024). Finally, a key innovation of this study lies in its inclusion of a postpartum follow-up assessment, allowing for the investigation of the long-term impact of the prenatal interventions on maternal wellbeing. To the best of our knowledge, no previous studies have explored the postpartum effects of prenatal relaxation interventions. This study represents an important initial step in understanding the enduring benefits of these types of interventions for maternal mental health.

This study also has some limitations that should be acknowledged. First, not all the participants completed all questionnaires and weekly diaries. Indeed, despite our efforts in collecting mothers’ responses at the three different timepoints, there was a significant drop-out in the questionnaire data, especially at t2, which may have introduced some bias into the findings and limited the statistical power to detect significant differences in some outcomes. Secondly, measures of perinatal outcomes considered in the study are limited. Future studies should expand the range of outcomes for example by including temperament, which has been reported to be sensitive to postnatal music interventions (e.g., Lejeune et al., 2019). Third, while participants were provided with a selection of songs and audio tracks, and those in the Active Protocol group were also allowed to expand this selection based on their own preferences, individual preferences were not comprehensively assessed and taken into consideration. This could have influenced in particular the effectiveness of the Active Protocol, as individual musical preferences may significantly impact maternal engagement and enjoyment with the prescribed activities. Finally, the reliance on self-report measures for assessing maternal wellbeing and attachment may be subject to inherent limitations, such as social desirability and recall biases, that may constraint speculations on symptom-level mental-health trajectories, emotional experience and transition to parenthood over the perinatal period.

In conclusion, the present study provides valuable insights into the shared and distinct effects of passive and active prenatal interventions on maternal and foetal wellbeing, and point to the importance of tailoring intervention strategies based on individual preferences and perinatal stage.

## Data Availability

The raw data supporting the conclusions of this article will be made available by the authors without undue reservation.

## Footnotes

^1^Qualtrics, Provo, UT, USA, https://www.qualtrics.com.
