# Supplementary material for: Exploring shared and unique benefits of passive and active prenatal intervention protocols on maternal wellbeing and neonatal outcomes: a combined quali-quantitative approach
Source: Front Psychol. 2025 Apr 29;16:1553946. doi: 10.3389/fpsyg.2025.1553946 (PMC12089647; doi:10.3389/fpsyg.2025.1553946)
Supplement: Supplementary file 1 [file Supplementary_file_1.docx]

**SUPPLEMENTARY INFORMATION S1**


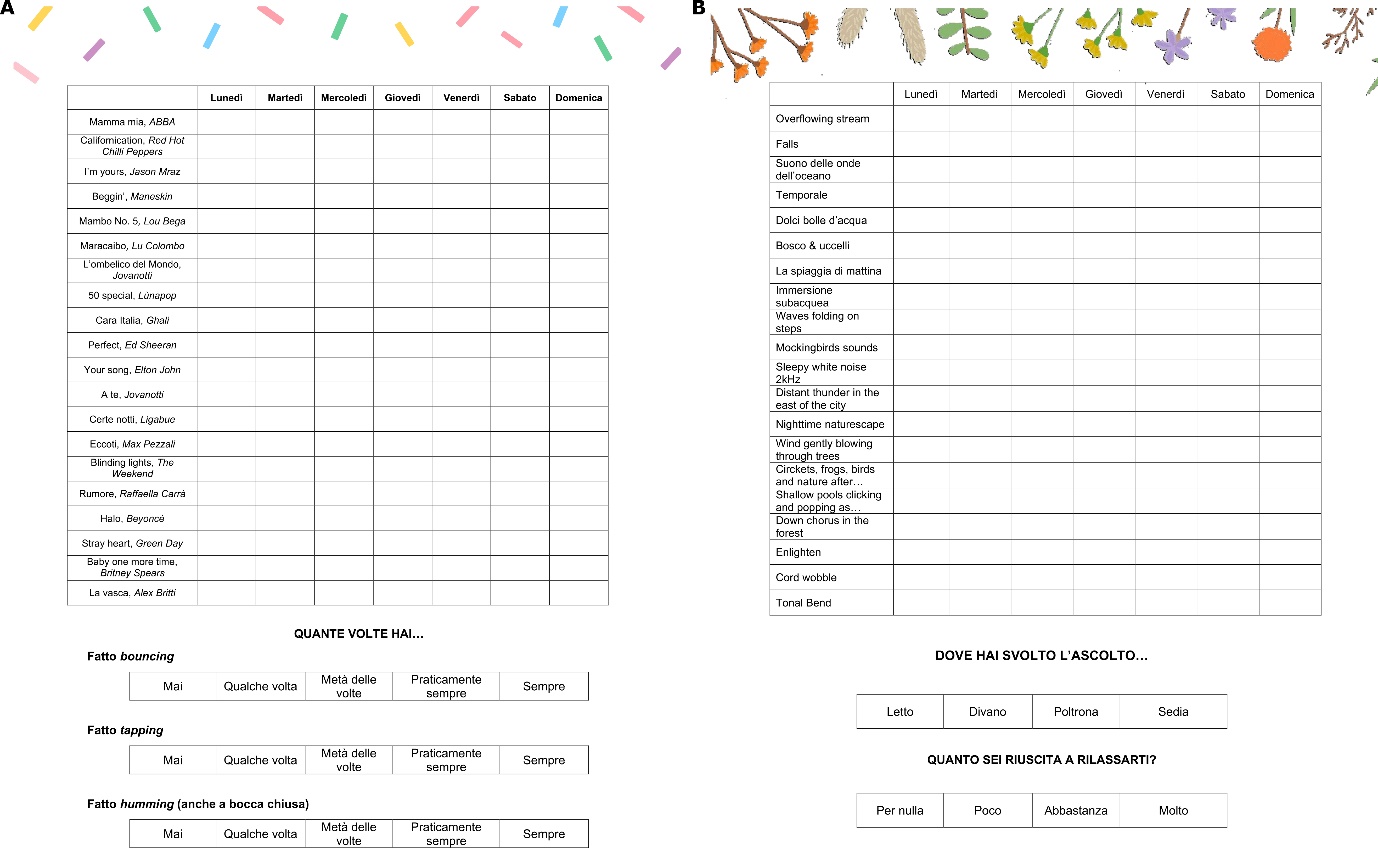


**Fig. S1.** Pages of the weekly diaries that expectant mothers were asked to fill, for both the Active Protocol (panel A) and Passive Protocol (panel B) groups. The frequency of intervention sessions was derived by summing the X put on the tables across all weeks of stimulation. For the Active Protocol group a total score of “Active Engagement” was calculated by summing the scores across the three questions regarding humming, tapping and bouncing (at the bottom of panel A) and averaging these scores across all the weeks of stimulation. For the Passive Protocol group, a total “Relaxation Engagement” score was calculated by averaging the weekly scores assessing the level of relaxation during the sessions (at the bottom of panel B).
