# Supplementary material for: Exploring shared and unique benefits of passive and active prenatal intervention protocols on maternal wellbeing and neonatal outcomes: a combined quali-quantitative approach
Source: Front Psychol. 2025 Apr 29;16:1553946. doi: 10.3389/fpsyg.2025.1553946 (PMC12089647; doi:10.3389/fpsyg.2025.1553946)
Supplement: Supplementary file 2 [file Supplementary_file_2.docx]

**SUPPLEMENTARY INFORMATION S2**

**Soundtrack selection**

Active Protocol

An initial pool of 40 songs (20 Italian, 20 English) was selected based on their distinct and easily discernible rhythm. To ensure participant input in the song selection process, an independent sample of 92 pregnant women were asked to rate the appropriateness of these songs for performing the activities prescribed by the Active Protocol (humming, tapping, and bouncing). To this end, the 40 songs were randomly divided into two groups, creating two separate surveys, each including 10 Italian songs and 10 English songs. Fifty-seven women filled the first survey, while 35 completed the second survey. Each survey included the following question: “How easy would it be for you to move, hum and tap your belly while listening to the song XXX"? Participants rated each song on a 5-point Likert scale (1 = “not easy” to 5 = “very easy”). To enhance understanding, an illustrative video demonstrating a pregnant woman performing the prescribed activities (humming, moving, and tapping) to the rhythm of music was provided.The top 10 highest-rated songs from each survey were included in the final playlist for the Active Protocol.

*List of selected songs*

1. Mamma mia, ABBA
2. Californication, Red Hot Chilli Peppers
3. I’m yours, Jason Mraz
4. Beggin’, Maneskin
5. Mambo No. 5, Lou Bega
6. Maracaibo, Lu Colombo
7. L’ombelico del Mondo, Jovanotti
8. 50 special, Lùnapop
9. Cara Italia, Ghali
10. Perfect, Ed Sheeran
11. Your song, Elton John
12. A te, Jovanotti
13. Certe notti, Ligabue
14. Eccoti, Max Pezzali
15. Blinding lights, The Weekend
16. Rumore, Raffaella Carrà
17. Halo, Beyoncé
18. Stray heart, Green Day
19. Baby one more time, Britney Spears
20. La vasca, Alex Britti

Passive Protocol

The 20 relaxing sounds were chosen from various Spotify relaxing playlists, to create a balanced combination of different nature sounds (e.g., waterfalls, river, sea waves, birds songs, wind) and white noises.

*List of selected soundtracks*

1. Overflowing stream
2. Falls
3. Suono delle onde dell’oceano
4. Temporale
5. Dolci bolle d’acqua
6. Bosco & Uccelli
7. La spiaggia di mattina
8. Immersione subacquea
9. Waves folding on steps
10. Mockingbirds sounds
11. Sleepy white noise 2kHz
12. Distant thunder in the east of the city
13. Nighttime naturescape
14. Wind gently blowing through trees
15. Crickets, frogs, birds and nature after…
16. Shallow pools clicking and popping as…
17. Down chorus in the forest
18. Enlighten
19. Cord wobble
20. Tonal Bend
