# Supplementary material for: Exploring shared and unique benefits of passive and active prenatal intervention protocols on maternal wellbeing and neonatal outcomes: a combined quali-quantitative approach
Source: Front Psychol. 2025 Apr 29;16:1553946. doi: 10.3389/fpsyg.2025.1553946 (PMC12089647; doi:10.3389/fpsyg.2025.1553946)
Supplement: Supplementary file 3 [file Supplementary_file_3.docx]

**SUPPLEMENTARY INFORMATION S3**

**Thematic analysis on participants’ emotional experiences**

Emotional experiences were assessed through a single open-ended question in the weekly diary: “*What emotions and sensations did you experience while carrying out the activity?”*. Thematic analysis of these qualitative data was conducted by two independent researchers, with supervision from a third researcher. The analysis proceeded in three phases. The first step involved becoming familiar with the data: both researchers independently reviewed all participants' responses and identified the most prominent emerging contents within each group. In the second phase, the researchers, along with the supervisor, compared and discussed the identified contents and grouped them into "themes". The last phase resulted in the development of a coding scheme consisting of four major themes: positive emotions, mother-foetus relation, daily coping and negative emotions. A detailed description of these themes is provided in Table S3. Subsequently, the two researchers independently coded how many times each theme appeared in each participant’s diary. Inter-rater reliability was assessed for each of the four themes using Inter-rater correlation coefficients (ICC) for single raters. demonstrating high levels of agreement across the themes (mean ICC = .76, range = .63-.84, all *ps* < .001). For subsequent analyses, data from one of the two coders were randomly selected.

**Table S3.** Coding structure of the diary thematic analysis.

| **Themes** | **Description** | **Examples** |
| --- | --- | --- |
| Positive emotions | Feeling of joy, fun, happiness, cheerfulness, well-being, serenity, positivity. | “I felt joy and fun” |
| Mother-foetus relation | Emotional and Psychological Connections to the Foetus, often expressed through mentalization of the foetus and fantasizing about the child’s future physical appearance and personality. | “I feel in contact with her”; “She likes the song as she moves a lot during the listening”; “I was thinking about what he's going to do when he hears the songs I played for him this week”. |
| Daily coping | Feeling of distancing from everyday life, finding an outlet, relaxing. | “We relaxed while listening to the sounds”; "I distanced myself from routines and set aside problems and worries"; "The sounds transported me to an evening in the mountains." |
| Negative emotions | Tiredness and physical fatigue in carrying out the activity, irritability, sadness, anxiety and fear about childbirth and complications | "On Friday I was more agitated and I couldn’t relax”; "I'm a little anxious about the childbirth" |
