# Supplementary material for: Exploring shared and unique benefits of passive and active prenatal intervention protocols on maternal wellbeing and neonatal outcomes: a combined quali-quantitative approach
Source: Front Psychol. 2025 Apr 29;16:1553946. doi: 10.3389/fpsyg.2025.1553946 (PMC12089647; doi:10.3389/fpsyg.2025.1553946)
Supplement: Supplementary file 4 [file Supplementary_file_4.docx]

**Effects of maternal demographics and mental health conditions at t0 on adherence to the protocol**

To test whether maternal demographics and mental health conditions at t0 impacted the adherence to the protocol activities (i.e., number of intervention sessions, active engagement and passive engagement) we performed a series of linear regressions with number of intervention sessions, active engagement or passive engagement as dependent variables and maternal age, education, employment status, Socio-Economic Status (SES), years of relationship, STAI total scores at t0 or EPDS total score at t0, as independent variables.

Results showed that the employment status significantly predicted the engagement with the activities of the Active protocol, *b* = .69, *t*(65) = 2.11, *p* = .038. A similar marginally significant trend was found for the SES, *b* = .24, *t*(65) = 1.84, *p* = .07. Additionally, we found that the STAI total scores at t0 negatively affected the engagement with Passive protocol, *b* = .01, *t*(41) = 2.49, *p* = .017, and a similar trend was observed for the EPDS total score at t0, *b* = .02, *t*(41) = 1.93, *p* = .06. No other significant effects were found, *p* > .08.


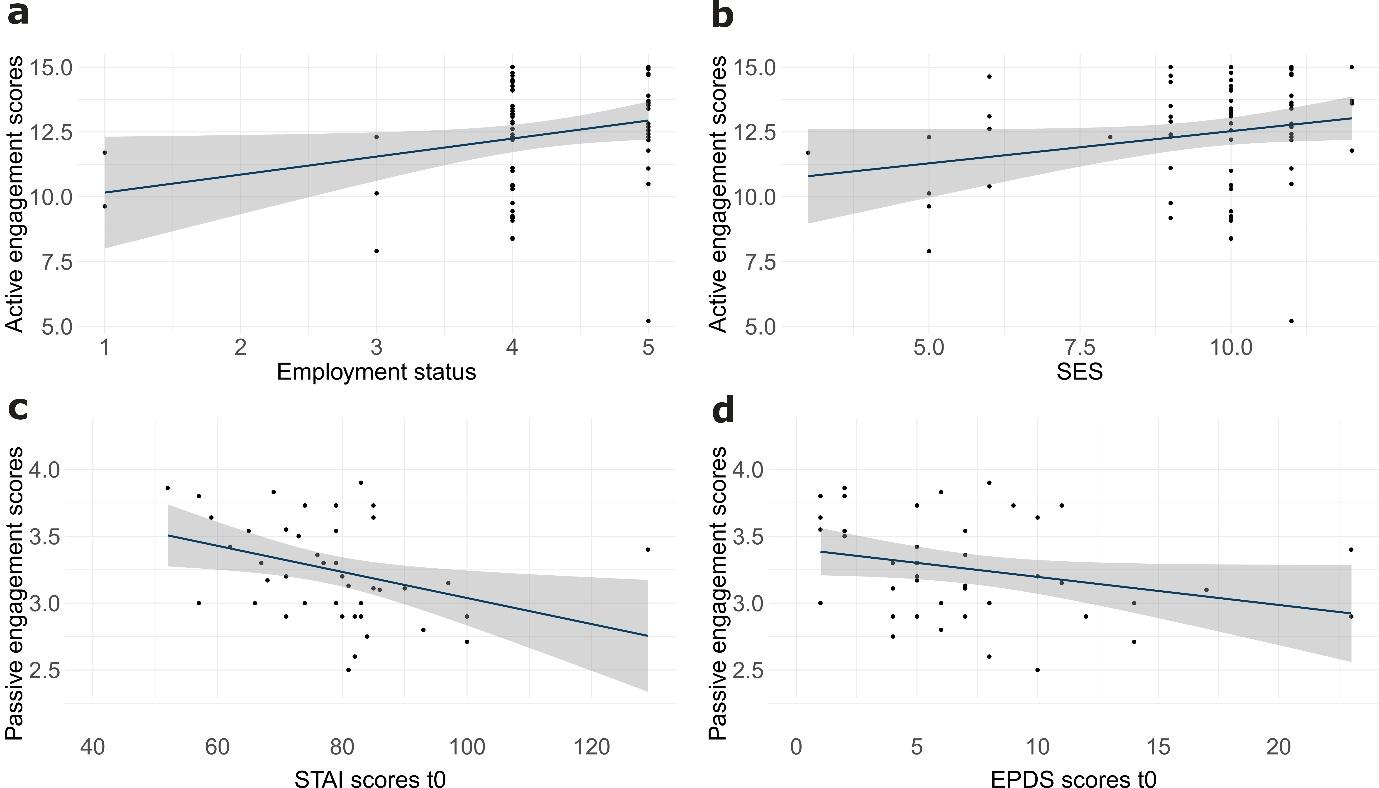


**Figure S4.** Panel a depicts the association between the Employment status and Active Engagement scores in the Active group. Panel b illustrates the association between the Socio-Economic Status and the Active Engagement scores in the Active Protocol group. Panel c depicts the association between STAI total scores at t0 and the Passive Engagement scores in the Passive group. Panel d shows the association between EPDS total scores at t0 and the Passive Engagement scores in the Passive group. Grey areas display confidence intervals.
